# Medical mobile technologies – what is needed for a sustainable and scalable implementation on a global scale?

**DOI:** 10.1080/16549716.2017.1344046

**Published:** 2017-08-25

**Authors:** Johan Lundin, Guy Dumont

**Affiliations:** ^a^ Institute for Molecular Medicine Finland – FIMM, University of Helsinki, Helsinki, Finland; ^b^ Department of Public Health Sciences, Karolinska Institutet, Stockholm, Sweden; ^c^ Department of Electrical and Computer Engineering, University of British Columbia, Vancouver, Canada; ^d^ Stellenbosch Institute for Advanced Study (STIAS), Wallenberg Research Centre at Stellenbosch University, Stellenbosch, South Africa

**Keywords:** mHealth for ImprovedAccess and Equity in Health Care, Mobile health, global health, technological innovations, diagnostic imaging, health resources

## Abstract

Current advances within medical technology show great potential from a global health perspective. Inexpensive, effective solutions to common problems within diagnostics, medical procedures and access to medical information are emerging within almost all fields of medicine. The innovations can benefit health care both in resource-limited and in resource-rich settings. However, there is a big gap between the proof-of-concept stage and implementation. This article will give examples of promising solutions, with special focus on mobile image- and sensor-based diagnostics. We also discuss how technology and frugal innovations could be made sustainable and widely available. Finally, a list of critical factors for success is presented, based on both our own experiences and the literature.

## Background

### Frugal mobile health innovations

Advances within the fields of connected and mobile health (mHealth) have recently generated a series of promising medical technologies that address global health-related challenges. Innovations include inexpensive, effective solutions to common problems within diagnostics, medical procedures and access to medical information often with minimal use of resources [1]. These so-called frugal innovations have often been sparked by global health initiatives, donators and non-governmental organizations (NGOs) and typically can benefit health care both in resource-limited and in higher-income settings.

Inexpensive, mass-produced components from the mobile and microelectronics industry, 3D printing, miniaturization, artificial intelligence and lab-on-a-chip techniques allow for unexpected, low-cost and point-of-care solutions. Task-shifting, where for example diagnostic devices and tests can be used by less-skilled persons or patients themselves, can improve access to diagnostics in a global perspective. The novel mobile solutions enable health care to be provided wherever people are, not only in health-care facilities.

However, most of the innovations still remain at a proof-of-concept stage or are limited to pilot studies, the so-called ‘pilotitis’ syndrome; see Sundin et al. [2] for an analysis of a number of failure modes for mHealth projects. Examples include the Ghana-based HealthKeepers, an mHealth organization that failed due to its inability to attract external funding, and the Zambia-based Mwana, which failed due to underestimation of the computing power required by its servers when scaling. This rate of failures is so alarming that some countries such as Uganda refuse to engage in any new mHealth projects. There is also the question whether more of already existing medical technologies could be made cheaper and accessible in resource-limited settings and whether the advances in mobile technologies really have been fully utilized.

### Unleash the global potential of digital medicine and connected health

In this article, we bring up aspects on how the huge advances in connectivity, access to information and computational capacity could have an even bigger impact on health care and how the potential could be unleashed within a global health setting. The use of mobile technologies could improve the quality of services and access to health care, particularly in underserved rural areas [3].

Connected health solutions, electronic medical records, geographical positioning systems, sensors and cloud computing can contribute to more efficient health care and are part of the digitalization of medicine. Future perspectives include an increasing use of artificial intelligence (AI) and machine learning (ML) in computer-assisted clinical decision-support systems (CDSS). AI will provide support and intelligence augmentation rather than replace the human expert. AI and CDSS will assist experts to perform better and through machine learning make use of previously collected and shared information. Surveillance and epidemiology will greatly benefit from improved connectivity and processing of already existing big data, connecting distributed data sources and location information. Mobile connectivity also provides efficient channels for distribution of information, reminders and follow-up to monitor the effects of treatment.

### Image-based diagnostics

A specific field of mobile medical technologies relates to the use of imaging. A picture is worth a thousand words, which clearly also holds true within mobile health. A large number of mobile health solutions have been presented within this field, including applications for emergency medicine, surgery, otolaryngology, ophthalmology, dermatology, radiology, pathology and general medicine [4]. Specific applications such as burn injury consultation [5], oral cancer diagnostics [6], diagnosis of middle-ear infections [7], refraction tests and cataract assessment (Eyenetra, Somerville, MA), skin cancer detection (SkinVision, SkinVision BV, Amsterdam, Netherlands and MoleScope, MetaOptima, Vancouver, BC) and parasite and cancer microscopy-based diagnosis [8–10] to name a few. A series of portable ultrasound systems are available that can display the image on portable devices, providing rugged low-cost systems that do not compromise the image quality (VScan, GE Healthcare, Chicago, IL, and Clarius, Burnaby BC, Canada). Recently, a smartphone-based platform suitable for low-cost luminescence-based point-of-care (POC) diagnostics was developed at the University of British Columbia (UBC) using nanoparticles [11]. Nanoparticles that exhibit bright luminescence can be designed to interact with biological systems through control of their surface chemistry and spectroscopy, allowing visualization of biomarkers and thus providing new opportunities for developing diagnostic methods. Also simpler solutions, like taking photos of patient findings, paper documents, logbooks and x-ray films with a smartphone at the point-of-care and sending these through common messaging platforms (e.g. MMS, Whatsapp, Messenger etc.) for consultation, could be considered as part of image-based diagnostics [12,13].

### Sensor-based diagnostics

Also, other mobile light-based sensors like those included in pulse-oximeters or in near-infrared spectroscopy are closely related to the imaging applications. The Phone Oximeter^TM^ developed at UBC [14], approved as a Class II Medical device by Health Canada (Health Canada Medical Device Licence # 94,684) and commercialized (LionsGate Technologies, Kenek O2, Vancouver, Canada; http://www.lgtmedical.com), provides medical-grade oxygen saturation and heart-rate measurements at a fraction of the cost of standard medical oximeters. Such a device enables further versatility of medical mobile diagnostics particularly when coupled with predictors of risks based on predictive analytics. This is the case with the *PIERS on the Move* application [15,16] for risk assessment in pre-eclampsia that is currently being tested on thousands of pregnant women in Mozambique and Pakistan as part of a large initiative funded by the Bill & Melinda Gates Foundation (https://pre-empt.cfri.ca, last visited 10 November 2016). More recently, a portable smartphone-connected near-infrared spectroscopy (NIRS) device has been proposed [17] that will allow for low-cost monitoring of brain, muscle and splanchnic hemodynamics. It is also now possible to monitor and collect a number of vital signs using a smartphone with accuracy comparable to more expensive medical devices, e.g. ECG (AliveCor, San Francisco, CA, uber Diagnostics Vijayanagar, India), blood pressure (AliveCor, LionsGate Technologies), temperature (LionsGate Technologies), while EEG systems are being developed, see e.g. DeLone and McLean [18].

### How to achieve sustainability and scalability?

Given all the promise of mobile medical technologies, there is a clear discrepancy between the number of presented and implemented innovations. There are obvious challenges in making technology and frugal innovations sustainable and widely available. Financing and raising awareness are essential parts of the process and needed to bring safe and effective solutions to the market more quickly. Currently organizations such as WHO, Foundation for Innovative New Diagnostics (FIND) and Program for Appropriate Technology in Health (PATH) can provide support, but sustainability requires financial planning with a sound value proposition as the basis for a sustainable business, effective partnerships, local processes and policy-making to also be successful.

Simplifying the process of designing and testing novel technologies is important, since the field is rapidly advancing and regulatory hurdles can be a challenge for both software and hardware companies. Eckman et al. propose a conceptual framework they call design thinking that engages key stakeholders right at the start of any mHealth project [19]. An efficient and early collaboration between academia, industry, non-governmental organizations and governments is needed for success. Micro-financing instruments can support the distribution of new products and through the whole process universities play a key role by providing scientific evaluation of novel technologies.

### Critical factors for success

Based on the literature [4,11,18,20–23] and our own experiences we have identified the following factors as critical in creating sustainable and scalable solutions within the field of mobile and connected health:

1. Align the new solution with a country’s existing infrastructure and health strategies as well as economics of health care within the government. Development and scaling of information and communication technologies (ICT) are largely driven by communication and commerce needs in general. To facilitate the improvement of mobile infrastructure in resource-limited settings mobile health solutions should be considered in a broader context and coupled with similar solutions for education, agriculture and small businesses.
2. Identify the real needs, understand the local settings, e.g. existing health care, mobile infrastructure language requirements, cultural practices, understand what motivates the end-user and what contributes to user satisfaction. Engage the community from the beginning and monitor the perceived usefulness closely.
3. Use agile product development, i.e. start the implementation and field tests as early as possible in the process. Get feedback from the end-users through rigorous usability studies and the perceived ease of use. Correct mistakes in design and usability early, perform rapid iterations of the solution and then redeploy the new version. This approach has proven highly effective as compared to traditional approaches where implementation happens late in the project.
4. Involve all stakeholders, including health ministry and telecommunications agencies, network operators, donors and end-users right from the start of the project. Describe benefits to both end-users and business partnerships, understand all partners’ success metrics. All stakeholders must be presented with a compelling value proposition to ensure sustainability.
5. Take scalability factors into consideration from the start. A business model is needed to ensure scaling, it cannot rely on short-term funding. A financially viable business is the only way to ensure perennity.
6. Financing of projects is typically a problem and seed money often comes from private philanthropists and donors. It should rather be mainstreamed and include industry, telecom companies, pharmaceutical companies and NGOs.
7. Promote the use of standards and integration with the local health information-management systems. Join global data and application repositories, promote joint collections of data and images. Within image-based diagnostics, access to large annotated image databases is instrumental both in the development of new algorithms and in validation of existing ones.
8. Evaluate feasibility and monitor the impact and cost efficiency of a new method, taking into account the local financial (e.g. different level of subsidy) and technical resources (e.g. mobile network coverage). Currently only a very small proportion of mobile health solutions are evaluated. There needs to be investment in the evaluation, both financial and human resources. Scale-up should be preceded by efficacy and effectiveness trials so that it is founded on an appropriate evidence base.

## Conclusions

Recent innovations within mobile and connected health show great promise for a number of global health-related diagnostic purposes. To implement, scale and sustain these innovations and solutions, a number of important factors need to be taken into account. Given the challenges, the authors are still convinced that if the above-mentioned critical steps are taken, a substantial number of mobile health innovations will move into clinical practice and wide use during the near future.

## Paper context

The extensive spread of mobile phones and mobile wireless networks globally has led to a series of innovations within health care that take advantage of the novel technologies and the connectivity. These innovations often enable low-cost solutions to medical diagnostics and improved access to information and decision-support at the point-of-care. The article gives a brief overview of recent mobile diagnostic technologies, summarizes challenges and lists a series of critical factors for a successful implementation and scale-up.
